# A Mobile App to Support Self-Management in Patients with Multiple Myeloma or Chronic Lymphocytic Leukemia: Pilot Randomized Controlled Trial

**DOI:** 10.2196/44533

**Published:** 2023-07-06

**Authors:** Matthew R LeBlanc, Thomas W LeBlanc, Qing Yang, Jennifer McLaughlin, Kerry Irish, Sophia K Smith

**Affiliations:** 1 School of Nursing University of North Carolina at Chapel Hill Chapel Hill, NC United States; 2 Lineberger Comprehensive Cancer Center University of North Carolina Chapel Hill, NC United States; 3 Duke Cancer Institute Durham, NC United States; 4 School of Medicine Duke University Durham, NC United States; 5 School of Nursing Duke University Durham, NC United States; 6 Pattern Health Durham, NC United States; 7 AdventHealth Cancer Services Asheville, NC United States; 8 Dempsey Centers for Quality Cancer Care Lewiston, ME United States

**Keywords:** chronic lymphocytic leukemia, distress, intervention, leukemia, mHealth, mobile application, multiple myeloma, post-traumatic stress, self-management, symptoms, treatment

## Abstract

**Background:**

Patients with blood cancer experience serious physical and emotional symptoms throughout their cancer journey.

**Objective:**

Building on previous work, we aimed to develop an app designed to help patients with multiple myeloma and chronic lymphocytic leukemia self-manage symptoms and test it for acceptability and preliminary efficacy.

**Methods:**

We developed our Blood Cancer Coach app with input from clinicians and patients. Our 2-armed randomized controlled pilot trial recruited participants from Duke Health and nationally in partnerships with the Association of Oncology Social Work, Leukemia and Lymphoma Society, and other patient groups. Participants were randomized to the attention control (Springboard Beyond Cancer website) arm or the Blood Cancer Coach app intervention arm. The fully automated Blood Cancer Coach app included symptom and distress tracking with tailored feedback, medication reminders and adherence tracking, multiple myeloma and chronic lymphocytic leukemia education resources, and mindfulness activities. Patient-reported data were collected at baseline, 4 weeks, and 8 weeks for both arms through the Blood Cancer Coach app. Outcomes of interest were global health (Patient Reported Outcomes Measurement Information System Global Health), posttraumatic stress (Posttraumatic Stress Disorder Checklist for DSM-5), and cancer symptoms (Edmonton Symptom Assessment System Revised). Among participants in the intervention arm, satisfaction surveys and usage data were used to evaluate acceptability.

**Results:**

Among 180 patients who downloaded the app, 49% (89) of them consented to participate and 40% (72) of them completed baseline surveys. Of those who completed baseline surveys, 53% (38) of them completed week 4 surveys (16 intervention and 22 control) and 39% (28) of them completed week 8 surveys (13 intervention and 15 control). Most participants found the app at least moderately effective at helping manage symptoms (87%), feeling more comfortable seeking help (87%), increasing awareness of resources (73%), and reported being satisfied with the app overall (73%). Participants completed an average of 248.5 app tasks over the 8-week study period. The most used functions within the app were medication log, distress tracking, guided meditations, and symptom tracking. There were no significant differences between the control and intervention arms at week 4 or 8 on any outcomes. We also saw no significant improvement over time within the intervention arm.

**Conclusions:**

The results of our feasibility pilot were promising in which most participants found the app to be helpful in managing their symptoms, reported satisfaction with the app, and that it was helpful in several important areas. We did not, however, find significantly reduced symptoms or improved global mental and physical health over 2 months. Recruitment and retention were challenging for this app-based study, an experience echoed by others. Limitations included a predominantly White and college educated sample. Future studies would do well to include self-efficacy outcomes, target those with more symptoms, and emphasize diversity in recruitment and retention.

**Trial Registration:**

ClinicalTrials.gov NCT05928156; https://clinicaltrials.gov/study/NCT05928156

## Introduction

Physical and emotional symptoms are common among cancer survivors due to their disease and its treatment and are particularly debilitating for those with blood cancers [1,2]. Blood cancer survivors experience serious physical (eg, insomnia and fatigue) and emotional (eg, worry and distress) symptoms throughout their cancer journey [3-5]. Among blood cancers, multiple myeloma (MM) and chronic lymphocytic leukemia (CLL) are the 2nd and 3rd most common types, respectively, and are considered incurable. MM and CLL have a chronic relapsing remitting course that often requires multiple lines of treatment [6,7]. This increases the potential for disease and treatment-related physical symptoms and emotional distress [6-9].

Interventions that target physical and emotional symptoms are lacking for blood cancer survivors. Due to the increasing use of technology, digital health solutions are becoming more commonplace and are helping to bridge the gap in services for underserved populations. For example, in 2021 it was estimated that 85% of adults in the United States own a smartphone, including most adults (61%, 65 years of age or older) and 80% of adults who are living in rural settings [10]. mHealth apps present exciting opportunities to augment patients’ disease self-management and meet needs wherever and whenever they arise in a way that is cost-effective, efficient, and convenient. Self-management apps have been developed and tested for those with diabetes, chronic lung disease, cardiovascular disease, and cancer, including Cancer Distress Coach which informed this study’s Blood Cancer Coach app [11-13].

Despite the growing use of mHealth apps, evidence of their effectiveness in cancer survivors remains sparse [14]. Therefore, the purpose of this study was to develop and test a blood cancer app aimed at augmenting self-management for adults living with MM and CLL for acceptability and preliminary efficacy.

## Methods

### App Development

Our Blood Cancer Coach app development was largely informed by the Cancer Distress Coach app developed previously by our research team [11]. Cancer Distress Coach is focused on education and self-management of cancer-related posttraumatic stress (PTS) symptoms and includes education, support resources, mindfulness exercises, and self-assessments [11]. With Cancer Distress Coach as our starting point, we interviewed patients with blood cancer and clinicians to determine how best to deliver a more targeted app to specifically meet their needs and challenges.

### Pilot Trial

Once developed, we aimed to test the app’s acceptability and preliminary efficacy through a 2-armed randomized pilot clinical trial. Trial participants were recruited from the Duke University Health System using the web-based patient portal (MyChart) to send email invitations. National recruitment was facilitated through partnerships with the Association of Oncology Social Work, Leukemia and Lymphoma Society, and private patient groups that were contacted through Facebook. Our eligibility criteria included having either a MM or CLL diagnosis, being at least 18 years of age, being able to read English, owning a smartphone (iPhone or Android), and basic computer and internet literacy. Recruitment and enrollment occurred between December 2020, and October 2021. Participants were followed for 8 weeks following enrollment.

Potential participants were provided access codes to download Blood Cancer Coach through Pattern Health’s iOS and Android platform app. The Blood Cancer Coach app was used to administer informed consent, randomize participants 1:1 to attention control (Springboard Beyond Cancer website) or the Blood Cancer Coach intervention arm, and collect self-reported data through surveys. Data were collected on intervention and control arm participants at baseline, and 4 and 8 weeks after study enrollment. Because our control arm was not a placebo, participants were aware of their group assignment.

### Measures

Demographic and clinical characteristics were self-reported at baseline. Outcomes were self-reported at baseline, week 4, and week 8 through the Blood Cancer Coach app. App usage was assessed at the end of the study period using Pattern Health app usage analytics. For those in the intervention arm, acceptability was measured using a perceived helpfulness and satisfaction survey administered at week 8. All data collection were done through the Blood Cancer Coach app.

Efficacy outcomes of interest were global health, posttraumatic stress, and cancer symptoms [15-17]. Global health was measured using the 10-item PROMIS (Patient Reported Outcomes Measurement Information System) Scale version 1.2-Global Health [18]. This scale results in summary global mental health (GMH) and global physical health (GPH) scores [18]. The Global Health scale is made up of 5-point Likert-type items. Scoring was done using HealthMeasures scoring service [19]. Like all PROMIS measures scores are transformed onto a T-score metric, in which 50 corresponds to the general population mean with SD of 10 [20]. Higher scores indicate better global physical and mental health [20].

Cancer symptoms were assessed using the 10-item Edmonton Symptom Assessment System Revised (ESAS-r), which measures 9 common cancer symptoms on a 0-10 rating scale [21,22]. A total symptom score was calculated for analysis by summing severity scores across symptoms. Higher scores correspond to higher symptom burden.

PTS symptoms were measured using the Posttraumatic Stress Disorder Checklist for DSM-5 (PCL-5) [17]. This 20 item instrument measures the severity of 20 symptoms of PTS on a 5-point Likert scale (0=not at all to 4=extremely). Item scores are summed to result in a continuous measure of PTS symptoms where higher scores indicate a higher burden of PTS symptoms. Previous psychometric evaluation revealed an internal consistency (α) of .94 and test-retest reliability (*r*) of 0.82 [17].

Acceptability was assessed using a study based on perceived helpfulness and satisfaction survey. Participants were asked to rate their overall satisfaction using the app and their perceptions of the helpfulness of different features of the app on a 5-point Likert scale. Participants were also invited to provide free-text feedback through two prompts: (1) what did you like best about the Blood Cancer Coach App? (2) How can we change Blood Cancer Coach to make it better? App usage was tracked by Pattern Health mobile app platform. A date and time stamped log was created when a user began a task (eg, logging a medication) that further indicated whether the task was completed.

### Analysis

Descriptive statistics were used to summarize participant characteristics across study arms. Results of the perceived helpfulness and satisfaction survey results were summarized with mean (SD). Further, the percentage of those endorsing Likert scale ratings of 3 (moderately satisfied or moderately helpful) was reported. Acceptability will be determined if more than 70% participants report overall satisfaction of moderate or better. Free text answers to the perceived helpfulness and satisfaction survey were narratively summarized to gain further insight into acceptability. We will also describe usage rates of the app overall and by task type.

Independent *t* tests were used to compare change from baseline to week 4 between intervention and control arms. Paired *t* tests were used to compare score changes from baseline to week 4, and from baseline to week 8 in participants of the intervention arm. Effect sizes were estimated using Cohen *d* [23].

### Ethics Approval

This study was approved by the Duke University IRB (Pro00105025). Patients reviewed study details and indicated their consent within the Blood Cancer Coach App. Patients were encouraged to contact our study team if they had any questions or concerns before consenting and at any time during the study. Our app development partner Pattern Health is approved by Duke University to participate in research activities including hosting sensitive patient health information. Patient health information collected through the app include, name, age in years, email address, and date of MM or CLL diagnosis. All data were encrypted in transit and at rest on Pattern Health servers. Data stored locally on participants’ mobile devices were encrypted by the Pattern Health App. Study team access to user data was password protected and limited to MRL, SKS, and JM. All data analyses were conducted on deidentified data. Patients did not receive compensation for this study.

## Results

### App Development

We interviewed 17 patients with blood cancer and 13 blood cancer clinicians to refine the Blood Cancer Coach mobile app. Our interviews used a previous app (Cancer Distress Coach) developed by the team as a starting point and explored what functionality would be helpful for the specific self-management needs of patients with blood cancer. These interviews resulted in several additions to the app which included feedback tailored to symptom severity, and the inclusion of a medication tracking feature with medication reminder notifications. Our Blood Cancer Coach app was developed in partnership with Pattern Health, a digital health platform provider, and was refined iteratively based on feedback from our clinician and cancer survivor partners [24].

The fully automated (no external human involvement) Blood Cancer Coach mobile app provides educational content on MM and CLL (treatments, symptom management, and available resources). Participants are prompted to record their emotional distress daily and their symptoms weekly through mobile phone notifications. Participants also have the ability to record distress and symptoms more often. A library of guided meditations is available to help participants manage distress. Tailored feedback is provided to encourage self-management and coach participants to reach out for support when appropriate. Symptom and distress graphs are generated to help participants understand and communicate patterns. The app also features custom medication reminders and a medication log to track adherence to cancer treatment and use of as needed medications.

### Pilot Trial

**Figure 1:**
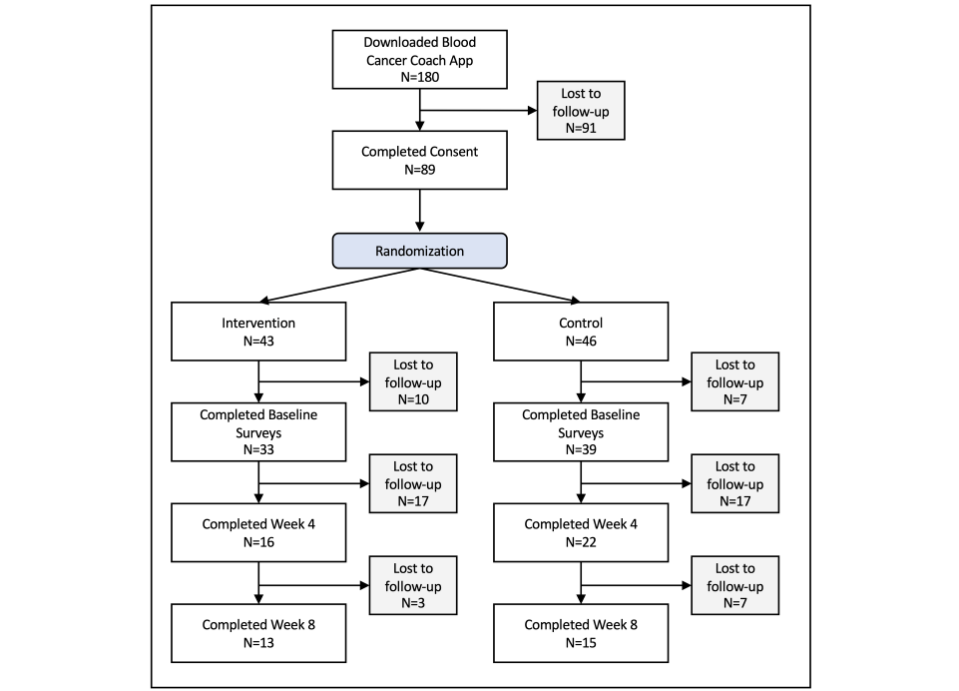
CONSORT (Consolidated Standards of Reporting Trials) subject flow diagram.

Among the 180 patients who downloaded the app, 49% (89/180) consented to participate, and 40% (72/180) completed baseline surveys. Of those who completed baseline surveys, 53% (38/180) completed week 4 surveys (16 intervention and 22 control) and 39% (28/180) completed week 8 surveys (13 intervention and 15 control; Figure 1). Demographics are reported for those who completed week 4 surveys (Table 1). Our sample was 50% (19/38) female, 92% (35/38) non-Hispanic White, 79% (30/38) college educated, and 8% (3/38) reported income less than US $30,000. Demographic and outcome measurements did not differ significantly at baseline between those who completed week 4 surveys and those who did not, except that those who completed baseline surveys but not week 4 surveys were on average 4.2 years older (*t*_70_=2.41, *P*=.02).

**Table 1:** Participant characteristics.

Characteristics		Total (N=38)		Control (N=22)		Intervention N=16
Age (years), mean (SD)		62.9 (7)		64.8 (6)		60.3 (7)
**Sex, n (%)**						
	Female	19 (50)	11 (50)		8 (50)	
	Male	19 (50)	11 (50)		8 (50)	
**Race, n (%)**						
	White	35 (92)	22 (100)		13 (81)	
	Black	3 (7)	0 (0)		3 (18)	
	Other	0 (0)	0 (0)		0 (0)	
**Ethnicity, n (%)**						
	Hispanic	1 (2)	1 (4)		0 (0)	
Partnered, n (%)		33 (87)		19 (86)		14 (87)
College graduate, n (%)		30 (79)		18 (81)		12 (75)
**Employment, n (%)**						
	Employed	18 (47)	6 (27)		12 (75)	
	Retired	16 (42)	12 (54)		4 (25)	
	Disabled	1 (2)	1 (4)		0 (0)	
	Homemaker	1 (2)	1 (4)		0 (0)	
**Income (US $), n (%)**						
	<30,000	3 (8)	3 (13)		0 (0)	
	30,000-59,999	5 (13)	3 (13)		2 (13)	
	60,000-89,999	9 (23)	4 (18)		5 (31)	
	>90,000	21 (55)	12 (55)		9 (56)	
Multiple myeloma, n (%)		14 (37)		8 (36)		6 (38)
Chronic lymphocytic leukemia, n (%)		24 (63)		14 (64)		10 (63)
Remission, n (%)		16 (42)		11 (50)		5 (31)
Current treatment, n (%)		17 (45)		10 (45)		7 (44)
**Past treatment, n (%)**						
	None	13 (34)	6 (27)		7 (44)	
	Surgery	2 (5)	2 (9)		0 (0)	
	Radiation therapy	2 (5)	1 (5)		1 (6)	
	Intravenous therapy	19 (50)	13 (59)		6 (38)	
	Oral therapy	18 (47)	11 (50)		7 (44)	
	Stem cell transplant	9 (23)	6 (27)		3 (19)	
Other cancer, n (%)		12 (32)		7 (32)		5 (31)

#### Acceptability Results

Of the 16 intervention arm participants, 15 participants completed our perceived helpfulness survey. Almost three quarters (n=11, 73%) reported at least moderate satisfaction with the app (Table 2). Additionally, most participants found the app at least moderately effective at helping manage symptoms (n=13, 87%), feeling more comfortable when seeking help (n=13, 87%), and increasing awareness of resources (n=11, 73%). Participants in the intervention arm completed an average of 148.5 (SD 118.6) app tasks during the 8-week study period and app usage ranged from 11 to 518 tasks completed. The most used functions within the app as measured by mean usage across participants were the medication log (mean 66.1, SD 76.3), distress tracking (mean 47.1, SD 25.5), and daily tips (mean 12.9, SD 21.5). Regarding the open-ended questions soliciting user satisfaction and perceived helpfulness, 27% (4/15) of participants cited the guided meditations and daily inspirational quotes as the best parts of the app, 20% (n=3) of them cited the ability to see how things change day by day, and 20% (3) of them said they appreciated the medication tracking and reminders. Three of 15 (20%) participants suggested changes in the way the app functioned, such as adding the ability to edit the previous day’s entries. Further, 2 of 15 (13%) participants mentioned that they themselves were not experiencing many symptoms and thought the app might be more helpful for those with higher burdens of physical symptoms and emotional distress.

**Table 2:** Perceived helpfulness and satisfaction^a^ (N=15).

Item		Mean (SD)	Endorsed moderately or greater, n (%)
Overall, how satisfied are you with Blood Cancer Coach?		3.27 (1.28)	11 (73)
**How helpful was Blood Cancer Coach in the following areas?**			
	Helping me find effective ways of managing my symptoms	3.33 (1.11)	13 (87)
	Helping me feel more comfortable in seeking support	3.07 (1.10)	13 (87)
	Helping me feel that there is something I can do about my symptoms	3.60 (1.18)	13 (87)
	Helping me track my symptoms	3.73 (1.39)	12 (80)
	Helping me to know when I am doing better or when I am doing worse	3.47 (1.40)	12 (80)
	Enhancing my knowledge of multiple myeloma or CLL^b^	3.33 (1.23)	12 (80)
	Helping me overcome the stigma of seeking mental health services	3.07 (1.28)	11 (73)
	Helping me better understand what I have been experiencing	3.27 (1.33)	11 (73)
	Increasing my access to additional resources	3.33 (1.29)	11 (73)
	Providing practical solutions to problems experience	3.13 (1.30)	10 (67)
	Providing a way for me to talk about what I have been experiencing	3.20 (1.37)	10 (67)
	Helping me learn about symptoms related to my multiple myeloma or CLL	2.93 (1.33)	9 (60)
	Helping me learn about treatments for my multiple myeloma or CLL	2.80 (1.47)	9 (60)

^a^Likert-scale values: 1=not at all; 2=slightly; 3=moderately; 4=very; 5=extremely.

^b^CLL: chronic lymphocytic leukemia.

#### Preliminary Efficacy Results

At week 4, there were no significant differences in change from baseline between control and intervention arms for any of our patient-reported outcomes (Table 3). Among those in the intervention arm, mean improvements in GPH from baseline to week 4 (mean 0.49, SD 3.5) and from baseline to week 8 (mean 0.23, SD 5.9) were nonsignificant (*P*=.59 and *P*=.17; Table 4). Similarly, improvements in GMH from baseline to week 4 (mean 0.16, SD 5.7) and baseline to week 8 (mean 2.2, SD 5.7) were nonsignificant (*P*=.91 and *P*=.19). Mean reductions in ESAS-r symptom scores from baseline to week 4 (mean –1.5, SD 6.8) and baseline to week 8 (mean –0.76, SD 5.6) were also nonsignificant (*P*=.39 and *P*=.63). Mean reductions in PCL-5 scores from baseline to week 4 (mean –0.69, SD 5.2) and baseline to week 8 (mean –1.5, SD 6.5) were nonsignificant as well (*P*=.61 and *P*=.41). Effect sizes, measured using Cohen *d*, ranged from 0.03 to 0.40 (Table 4).

**Table 3:** Differences in change from baseline to week 4, independent *t* test.

Reports	Score change intervention (n=16), mean (SD)	Score change control (n=22), mean (SD)	*t* test (*df*)	*P* value
Global physical health	0.49 (3.51)	0.76 (4.61)	0.20 (36)	.84
Global mental health	0.17 (5.70)	0.28 (3.98)	0.08 (36)	.94
Cancer symptoms	–1.50 (6.80)	–5.22 (10.18)	–1.27 (36)	.21
Posttraumatic stress	–0.69 (5.21)	–1.59 (3.02)	–0.67 (36)	.50

**Table 4:** Change over time within the intervention arm, paired *t* test.

Reports	Baseline to 4 weeks (n=16)			Baseline to 8 weeks (n=13)		
	Mean (SD)	Effect size^a^	*P* value	Mean (SD)	Effect size^a^	*P* value
Global physical health	0.49 (3.51)	0.14	.59	2.37(5.92)	0.40	.17
Global mental health	0.17 (5.70)	0.03	.91	2.18 (5.70)	0.38	.19
Cancer symptoms	1.50 (6.80)	0.22	.39	0.77 (5.64)	0.13	.63
Posttraumatic stress	0.69 (5.21)	0.13	.61	1.54 (6.50)	0.24	.41

^a^Effect size: Cohen *d.*

## Discussion

### Overview

In this study, we aimed to develop and pilot test a mobile health app to help patients with blood cancer self-manage their physical and emotional symptoms. Through an iterative process in partnership with clinicians and patients with blood cancer, we developed the Blood Cancer Coach mobile app for testing acceptability and preliminarily efficacy.

Participants in the intervention arm reported high levels of overall satisfaction (11/15, 73.3%) and reported that they found the app helpful in important domains we were hoping to impact, such as understanding, tracking, and managing symptoms (Table 2). We also noted a high level of engagement with the app as measured by tasks completed. These high levels of user satisfaction and engagement are evidence for our app’s acceptability and suggest that the Blood Cancer Coach app has the potential to help patients self-manage their MM- and CLL-related symptoms. On the other hand, high levels of study attrition are reason for concern and may suggest that the appeal of the app is limited to subpopulations of MM and CLL patients. In response to open-ended questions on our satisfaction survey, 2 participants indicated that they did not find the app useful and attributed this to the fact that they were experiencing low levels of symptoms and other issues. Perhaps, the app may not be useful or appealing to patients with low levels of physical and mental health concerns.

We found no significant effects on our outcomes of interest, either overtime in the intervention group, or between the intervention and control arms of the study. Negative efficacy results are not uncommon in mobile app studies. A recent systematic review of health behavior change mobile apps found that approximately 45% studies found no significant difference between mobile app users and comparator arms; furthermore, 31% of mobile app studies demonstrated some effectiveness in changing target health outcomes significantly more than comparator arms [25]. We believe there are several reasons for the nonsignificant findings among the outcomes of interest. For example, our sample size was quite small, and this pilot study was not powered to detect differences.

Unlike this study, a single arm pilot study of the Cancer Distress Coach app that served as our prototype found significant reductions in posttraumatic stress symptoms over 8 weeks [11]. Differences in app and study design may be instructive. The Cancer Distress Coach app was singularly focused on identifying and addressing emotional distress as measured by the posttraumatic stress disorder checklist, and it is possible that this more focused approach is more effective. Importantly, eligibility criteria required that participants have active symptoms of posttraumatic stress disorder. Responses to our free text survey question suggest that the app was not very helpful to those experiencing low levels of symptoms or distress. Further these responses also suggest the app may have had a positive effect on perceived self-management efficacy. Future studies should target participants with moderate to high levels of symptom burden and distress and include self-efficacy as an outcome.

We encountered several challenges and limitations while performing this study that are worth mentioning. Randomization resulted in suboptimal distribution of patient characteristics across trial arms. Of note, cancer symptoms as measured by the ESAS-r were substantially higher in the control arm across all study time points. Future studies with larger sample size and recruitment targeted toward patients with moderate to high levels of cancer symptoms would potentially address this limitation (Table 5). We also experienced significant attrition as only 39% of participants completed the week 8 surveys (ie, all planned data collection). These low response rates introduce potential bias if those who respond are systematically different from those who do not respond. Like other mHealth studies, our study sample was overwhelmingly White and highly educated (Table 1) [11,14]. This is problematic for several reasons, among them that racial minorities and those with lower socioeconomic status consistently report worse health outcomes than their White peers and those with higher socioeconomic status [26,27]. Our trials are not reaching the patient populations who might have the greatest need for emotional and physical symptom management, robbing us of evidence in these populations with high needs. A more targeted recruitment strategy focusing on underserved cancer patient populations is warranted.

**Table 5:** Patient-reported outcome scores across time^a^.

Reports		Baseline	Week 4	Week 8
**Global physical health, mean (SD)**				
	Intervention	46.02 (6.95)	46.51 (6.20)	48.92 (8.21)
	Control	46.32 (6.09)	47.08 (7.06)	46.47 (7.28)
**Global mental health, mean (SD)**				
	Intervention	49.49 (6.35)	49.66 (7.90)	52.52 (7.38)
	Control	46.59 (5.31)	46.87 (6.83)	46.68 (6.04)
**Cancer symptoms, mean (SD)**				
	Intervention	10.94 (7.63)	9.44 (7.47)	9.69 (8.27)
	Control	22.59 (15.24)	17.36 (11.90)	20.40 (13.74)
**Posttraumatic stress, mean (SD)**				
	Intervention	7.56 (6.21)	7.56 (6.21)	6.54 (6.60)
	Control	8.91 (9.26)	8.91 (9.26)	6.64 (6.10)

^a^Reported as mean (SD). Sample sizes for baseline and week 4 are as follows: intervention=16, control=22. Sample size for week 8 is as follows: intervention=13, control=15.

### Conclusions

Most treatment arm participants reported satisfaction with the app and that it was helpful in several important areas, though we did not find significantly improved GMH or GPH, cancer symptoms, or PTS over 2 months. Satisfaction survey results suggest the app may work best for those with higher symptom burden and that self-efficacy would be an important outcome to measure in future studies.

Recruitment and retention were challenging for this app-based study, an experience echoed by others [28]. Of particular concern is the lack of racially diverse and lower income participants, populations known to experience high levels of physical and emotional symptoms [26,27]. Future studies would do well to include self-efficacy outcomes, target those with moderate to high burdens of symptoms and distress, and emphasize diversity in recruitment and retention.

## Appendix

Multimedia Appendix 1 CONSORT-eHEALTH checklist (V 1.6.1).

## Abbreviations

CLL: chronic lymphocytic leukemia
ESAS-r: Edmonton Symptom Assessment System Revised
GMH: global mental health
GPH: global physical health
MM: multiple myeloma
PCL-5: Posttraumatic Stress Disorder Checklist for DSM-5
PROMIS: Patient Reported Outcomes Measurement Information System
PTS: posttraumatic stress

## Data Availability

The data sets generated and analyzed during this study are not publicly available but are available from the corresponding author on reasonable request.
